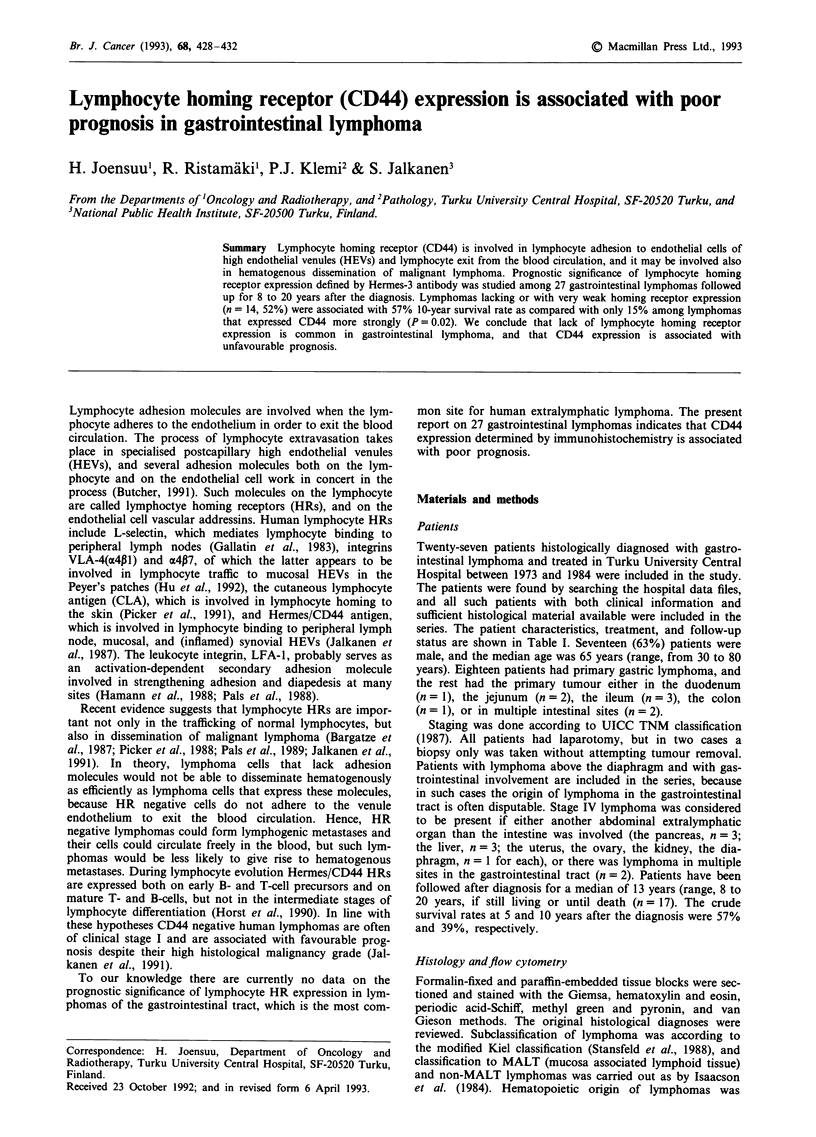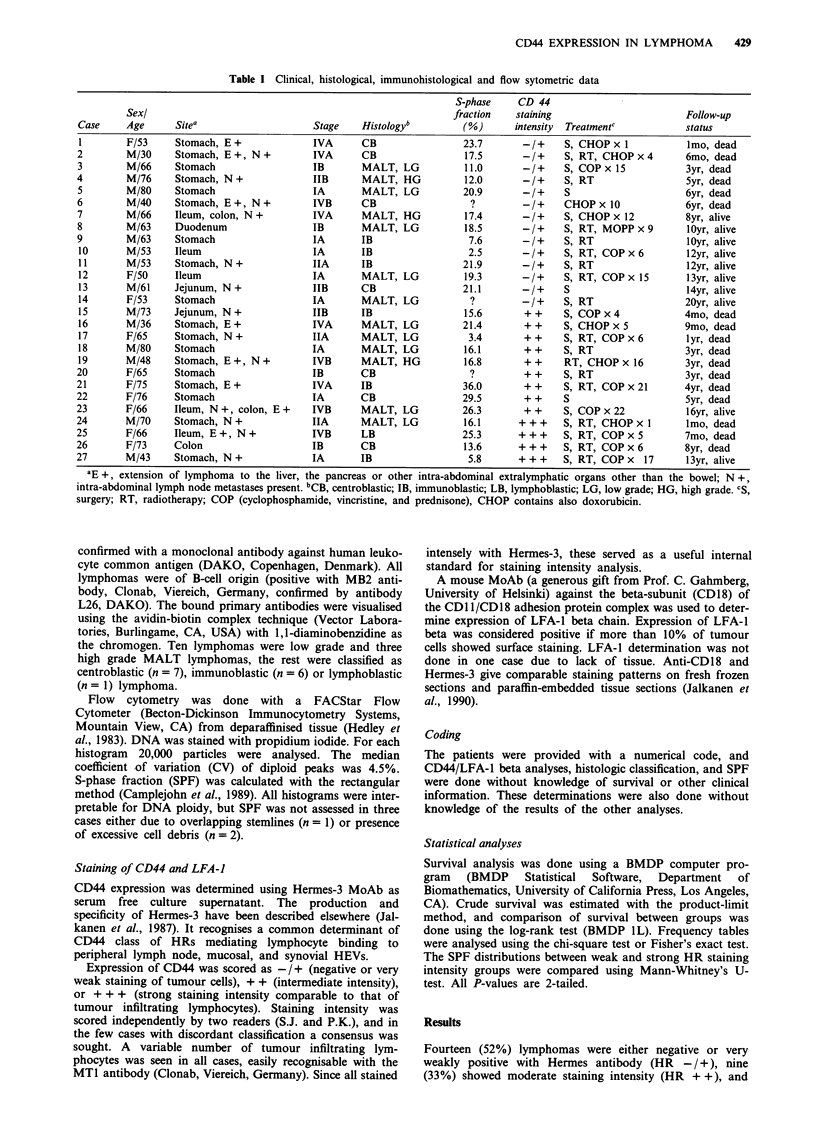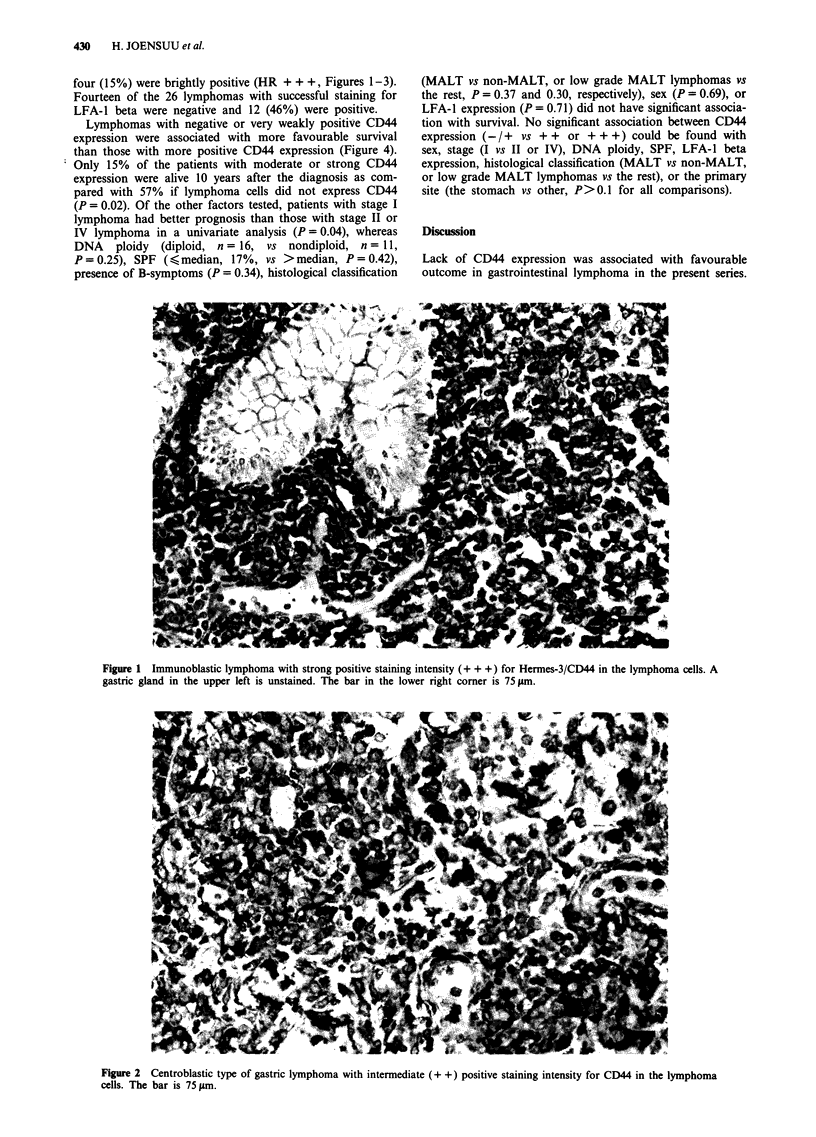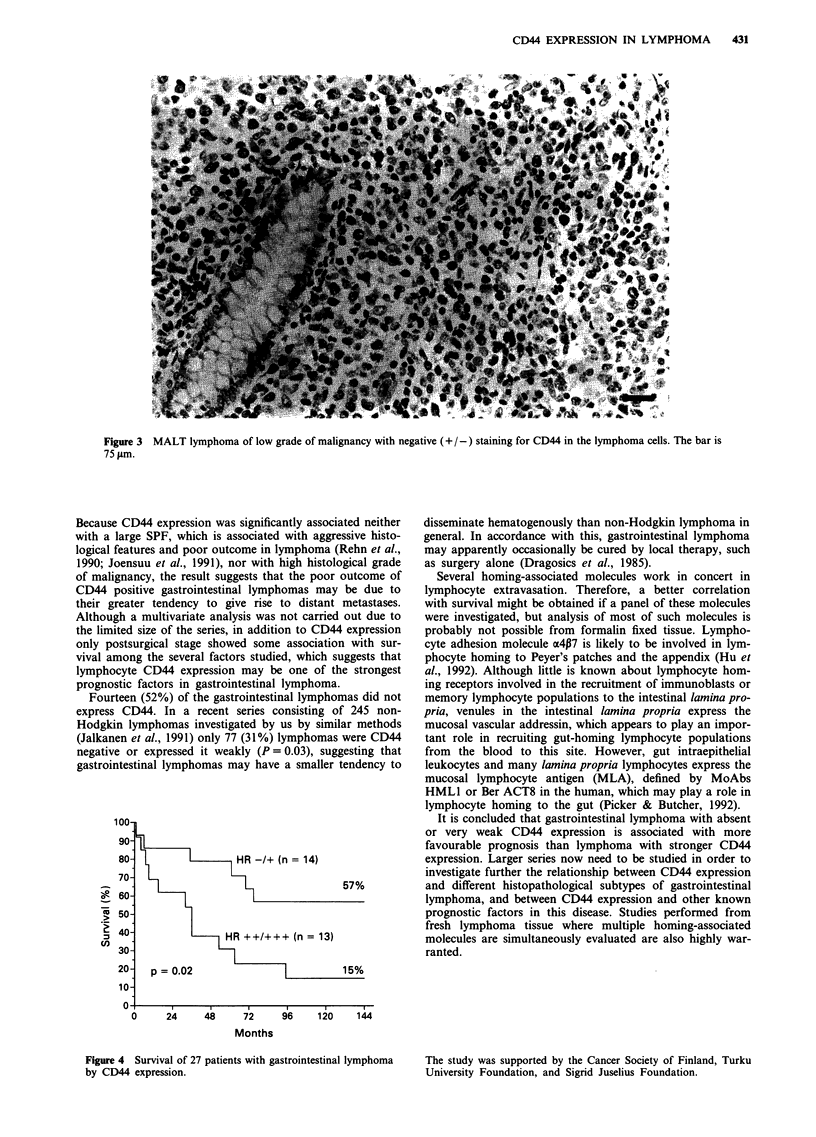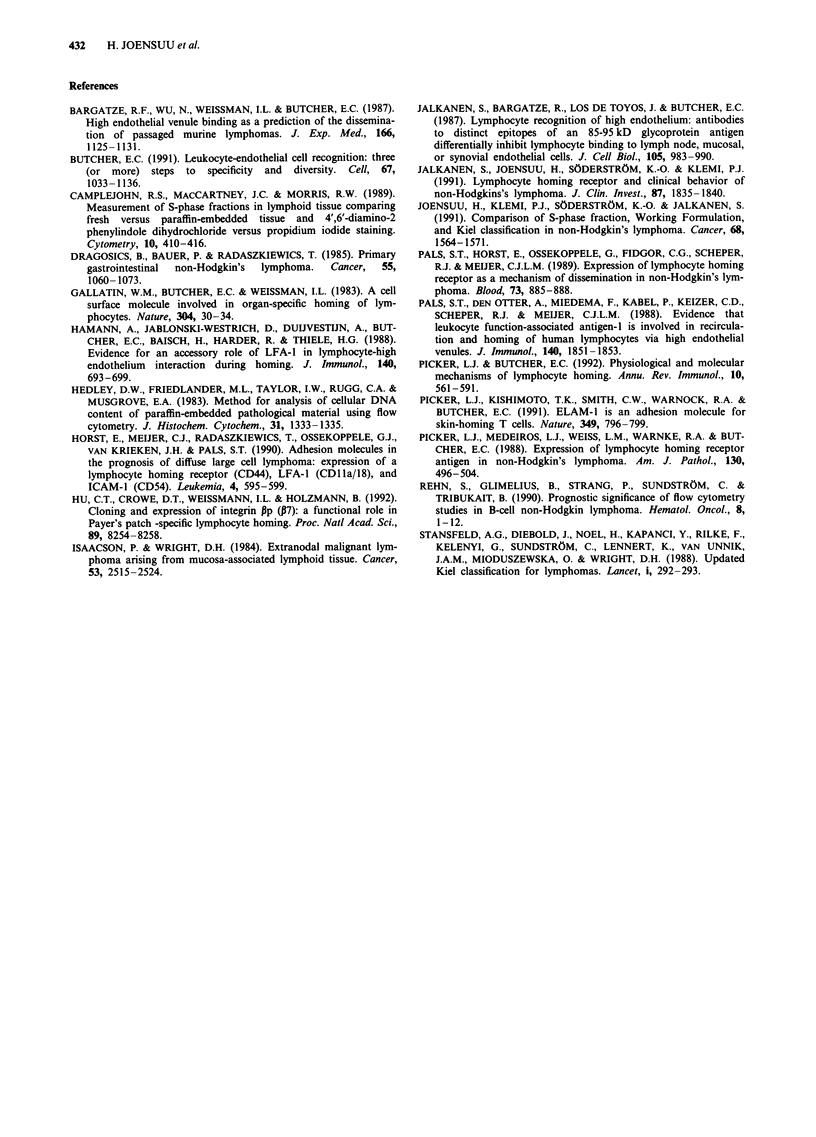# Lymphocyte homing receptor (CD44) expression is associated with poor prognosis in gastrointestinal lymphoma.

**DOI:** 10.1038/bjc.1993.354

**Published:** 1993-08

**Authors:** H. Joensuu, R. Ristamäki, P. J. Klemi, S. Jalkanen

**Affiliations:** Department of Oncology and Radiotherapy, Turku University Central Hospital, Finland.

## Abstract

**Images:**


Br. J. Cancer (1993), 68, 428–432 © Macmillan Press Ltd., 1993

### Lymphocyte homing receptor (CD44) expression is associated with poor prognosis in gastrointestinal lymphoma

H. Joensuu[1], R. Ristamäki[1], P.J. Klemi[2] & S. Jalkanen[3]

*From the Departments of [1]Oncology and Radiotherapy, and [2]Pathology, Turku University Central Hospital, SF-20520 Turku, and [3]National Public Health Institute, SF-20500 Turku, Finland.*

**Summary** Lymphocyte homing receptor (CD44) is involved in lymphocyte adhesion to endothelial cells of high endothelial venules (HEVs) and lymphocyte exit from the blood circulation, and it may be involved also in hematogenous dissemination of malignant lymphoma. Prognostic significance of lymphocyte homing receptor expression defined by Hermes-3 antibody was studied among 27 gastrointestinal lymphomas followed up for 8 to 20 years after the diagnosis. Lymphomas lacking or with very weak homing receptor expression ($n = 14$, 52%) were associated with 57% 10-year survival rate as compared with only 15% among lymphomas that expressed CD44 more strongly ($P = 0.02$). We conclude that lack of lymphocyte homing receptor expression is common in gastrointestinal lymphoma, and that CD44 expression is associated with unfavourable prognosis.

Lymphocyte adhesion molecules are involved when the lymphocyte adheres to the endothelium in order to exit the blood circulation. The process of lymphocyte extravasation takes place in specialised postcapillary high endothelial venules (HEVs), and several adhesion molecules both on the lymphocyte and on the endothelial cell work in concert in the process (Butcher, 1991). Such molecules on the lymphocyte are called lymphoctye homing receptors (HRs), and on the endothelial cell vascular addressins. Human lymphocyte HRs include L-selectin, which mediates lymphocyte binding to peripheral lymph nodes (Gallatin *et al.*, 1983), integrins VLA-4($\alpha 4 \beta 1$) and $\alpha 4 \beta 7$, of which the latter appears to be involved in lymphocyte traffic to mucosal HEVs in the Peyer's patches (Hu *et al.*, 1992), the cutaneous lymphocyte antigen (CLA), which is involved in lymphocyte homing to the skin (Picker *et al.*, 1991), and Hermes/CD44 antigen, which is involved in lymphocyte binding to peripheral lymph node, mucosal, and (inflamed) synovial HEVs (Jalkanen *et al.*, 1987). The leukocyte integrin, LFA-1, probably serves as an activation-dependent secondary adhesion molecule involved in strengthening adhesion and diapedesis at many sites (Hamann *et al.*, 1988; Pals *et al.*, 1988).

Recent evidence suggests that lymphocyte HRs are important not only in the trafficking of normal lymphocytes, but also in dissemination of malignant lymphoma (Bargatze *et al.*, 1987; Picker *et al.*, 1988; Pals *et al.*, 1989; Jalkanen *et al.*, 1991). In theory, lymphoma cells that lack adhesion molecules would not be able to disseminate hematogenously as efficiently as lymphoma cells that express these molecules, because HR negative cells do not adhere to the venule endothelium to exit the blood circulation. Hence, HR negative lymphomas could form lymphogenic metastases and their cells could circulate freely in the blood, but such lymphomas would be less likely to give rise to hematogenous metastases. During lymphocyte evolution Hermes/CD44 HRs are expressed both on early B- and T-cell precursors and on mature T- and B-cells, but not in the intermediate stages of lymphocyte differentiation (Horst *et al.*, 1990). In line with these hypotheses CD44 negative human lymphomas are often of clinical stage I and are associated with favourable prognosis despite their high histological malignancy grade (Jalkanen *et al.*, 1991).

To our knowledge there are currently no data on the prognostic significance of lymphocyte HR expression in lymphomas of the gastrointestinal tract, which is the most common site for human extralymphatic lymphoma. The present report on 27 gastrointestinal lymphomas indicates that CD44 expression determined by immunohistochemistry is associated with poor prognosis.

Correspondence: H. Joensuu, Department of Oncology and Radiotherapy, Turku University Central Hospital, SF-20520 Turku, Finland.

Received 23 October 1992; and in revised form 6 April 1993.

### Materials and methods

### *Patients*

Twenty-seven patients histologically diagnosed with gastrointestinal lymphoma and treated in Turku University Central Hospital between 1973 and 1984 were included in the study. The patients were found by searching the hospital data files, and all such patients with both clinical information and sufficient histological material available were included in the series. The patient characteristics, treatment, and follow-up status are shown in Table I. Seventeen (63%) patients were male, and the median age was 65 years (range, from 30 to 80 years). Eighteen patients had primary gastric lymphoma, and the rest had the primary tumour either in the duodenum ($n = 1$), the jejunum ($n = 2$), the ileum ($n = 3$), the colon ($n = 1$), or in multiple intestinal sites ($n = 2$).

Staging was done according to UICC TNM classification (1987). All patients had laparotomy, but in two cases a biopsy only was taken without attempting tumour removal. Patients with lymphoma above the diaphragm and with gastrointestinal involvement are included in the series, because in such cases the origin of lymphoma in the gastrointestinal tract is often disputable. Stage IV lymphoma was considered to be present if either another abdominal extralymphatic organ than the intestine was involved (the pancreas, $n = 3$; the liver, $n = 3$; the uterus, the ovary, the kidney, the diaphragm, $n = 1$ for each), or there was lymphoma in multiple sites in the gastrointestinal tract ($n = 2$). Patients have been followed after diagnosis for a median of 13 years (range, 8 to 20 years, if still living or until death ($n = 17$). The crude survival rates at 5 and 10 years after the diagnosis were 57% and 39%, respectively.

### *Histology and flow cytometry*

Formalin-fixed and paraffin-embedded tissue blocks were sectioned and stained with the Giemsa, hematoxylin and eosin, periodic acid-Schiff, methyl green and pyronin, and van Gieson methods. The original histological diagnoses were reviewed. Subclassification of lymphoma was according to the modified Kiel classification (Stansfeld *et al.*, 1988), and classification to MALT (mucosa associated lymphoid tissue) and non-MALT lymphomas was carried out as by Isaacson *et al.* (1984). Hematopoietic origin of lymphomas was

**Table I** Clinical, histological, immunohistological and flow sytometric data

| Case | Sex/ Age | Site[a] | Stage | Histology[b] | S-phase fraction (%) | CD 44 staining intensity | Treatment[c] | Follow-up status |
|---|---|---|---|---|---|---|---|---|
| 1 | F/53 | Stomach, E + | IVA | CB | 23.7 | −/+ | S, CHOP × 1 | 1mo, dead |
| 2 | M/30 | Stomach, E +, N + | IVA | CB | 17.5 | −/+ | S, RT, CHOP × 4 | 6mo, dead |
| 3 | M/66 | Stomach | IB | MALT, LG | 11.0 | −/+ | S, COP × 15 | 3yr, dead |
| 4 | M/76 | Stomach, N + | IIB | MALT, HG | 12.0 | −/+ | S, RT | 5yr, dead |
| 5 | M/80 | Stomach | IA | MALT, LG | 20.9 | −/+ | S | 6yr, dead |
| 6 | M/40 | Stomach, E +, N + | IVB | CB | ? | −/+ | CHOP × 10 | 6yr, dead |
| 7 | M/66 | Ileum, colon, N + | IVA | MALT, HG | 17.4 | −/+ | S, CHOP × 12 | 8yr, alive |
| 8 | M/63 | Duodenum | IB | MALT, LG | 18.5 | −/+ | S, RT, MOPP × 9 | 10yr, alive |
| 9 | M/63 | Stomach | IA | IB | 7.6 | −/+ | S, RT | 10yr, alive |
| 10 | M/53 | Ileum | IA | IB | 2.5 | −/+ | S, RT, COP × 6 | 12yr, alive |
| 11 | M/53 | Stomach, N + | IIA | IB | 21.9 | −/+ | S, RT | 12yr, alive |
| 12 | F/50 | Ileum | IA | MALT, LG | 19.3 | −/+ | S, RT, COP × 15 | 13yr, alive |
| 13 | M/61 | Jejunum, N + | IIB | CB | 21.1 | −/+ | S | 14yr, alive |
| 14 | F/53 | Stomach | IA | MALT, LG | ? | −/+ | S, RT | 20yr, alive |
| 15 | M/73 | Jejunum, N + | IIB | IB | 15.6 | + + | S, COP × 4 | 4mo, dead |
| 16 | M/36 | Stomach, E + | IVA | MALT, LG | 21.4 | + + | S, CHOP × 5 | 9mo, dead |
| 17 | F/65 | Stomach, N + | IIA | MALT, LG | 3.4 | + + | S, RT, COP × 6 | 1yr, dead |
| 18 | M/80 | Stomach | IA | MALT, LG | 16.1 | + + | S, RT | 3yr, dead |
| 19 | M/48 | Stomach, E +, N + | IVB | MALT, HG | 16.8 | + + | RT, CHOP × 16 | 3yr, dead |
| 20 | F/65 | Stomach | IB | CB | ? | + + | S, RT | 3yr, dead |
| 21 | F/75 | Stomach, E + | IVA | IB | 36.0 | + + | S, RT, COP × 21 | 4yr, dead |
| 22 | F/76 | Stomach | IA | CB | 29.5 | + + | S | 5yr, dead |
| 23 | F/66 | Ileum, N +, colon, E + | IVB | MALT, LG | 26.3 | + + | S, COP × 22 | 16yr, alive |
| 24 | M/70 | Stomach, N + | IIA | MALT, LG | 16.1 | + + + | S, RT, CHOP × 1 | 1mo, dead |
| 25 | F/66 | Ileum, E +, N + | IVB | LB | 25.3 | + + + | S, RT, COP × 5 | 7mo, dead |
| 26 | F/73 | Colon | IB | CB | 13.6 | + + + | S, RT, COP × 6 | 8yr, dead |
| 27 | M/43 | Stomach, N + | IA | IB | 5.8 | + + + | S, RT, COP × 17 | 13yr, alive |

[a]E +, extension of lymphoma to the liver, the pancreas or other intra-abdominal extralymphatic organs other than the bowel; N +, intra-abdominal lymph node metastases present. [b]CB, centroblastic; IB, immunoblastic; LB, lymphoblastic; LG, low grade; HG, high grade. [c]S, surgery; RT, radiotherapy; COP (cyclophosphamide, vincristine, and prednisone), CHOP contains also doxorubicin.

confirmed with a monoclonal antibody against human leukocyte common antigen (DAKO, Copenhagen, Denmark). All lymphomas were of B-cell origin (positive with MB2 antibody, Clonab, Viereich, Germany, confirmed by antibody L26, DAKO). The bound primary antibodies were visualised using the avidin-biotin complex technique (Vector Laboratories, Burlingame, CA, USA) with 1,1-diaminobenzidine as the chromogen. Ten lymphomas were low grade and three high grade MALT lymphomas, the rest were classified as centroblastic ($n = 7$), immunoblastic ($n = 6$) or lymphoblastic ($n = 1$) lymphoma.

Flow cytometry was done with a FACStar Flow Cytometer (Becton-Dickinson Immunocytometry Systems, Mountain View, CA) from deparaffinised tissue (Hedley *et al.*, 1983). DNA was stained with propidium iodide. For each histogram 20,000 particles were analysed. The median coefficient of variation (CV) of diploid peaks was 4.5%. S-phase fraction (SPF) was calculated with the rectangular method (Camplejohn *et al.*, 1989). All histograms were interpretable for DNA ploidy, but SPF was not assessed in three cases either due to overlapping stemlines ($n = 1$) or presence of excessive cell debris ($n = 2$).

### *Staining of CD44 and LFA-1*

CD44 expression was determined using Hermes-3 MoAb as serum free culture supernatant. The production and specificity of Hermes-3 have been described elsewhere (Jalkanen *et al.*, 1987). It recognises a common determinant of CD44 class of HRs mediating lymphocyte binding to peripheral lymph node, mucosal, and synovial HEVs.

Expression of CD44 was scored as −/+ (negative or very weak staining of tumour cells), + + (intermediate intensity), or + + + (strong staining intensity comparable to that of tumour infiltrating lymphocytes). Staining intensity was scored independently by two readers (S.J. and P.K.), and in the few cases with discordant classification a consensus was sought. A variable number of tumour infiltrating lymphocytes was seen in all cases, easily recognisable with the MT1 antibody (Clonab, Viereich, Germany). Since all stained intensely with Hermes-3, these served as a useful internal standard for staining intensity analysis.

A mouse MoAb (a generous gift from Prof. C. Gahmberg, University of Helsinki) against the beta-subunit (CD18) of the CD11/CD18 adhesion protein complex was used to determine expression of LFA-1 beta chain. Expression of LFA-1 beta was considered positive if more than 10% of tumour cells showed surface staining. LFA-1 determination was not done in one case due to lack of tissue. Anti-CD18 and Hermes-3 give comparable staining patterns on fresh frozen sections and paraffin-embedded tissue sections (Jalkanen *et al.*, 1990).

### *Coding*

The patients were provided with a numerical code, and CD44/LFA-1 beta analyses, histologic classification, and SPF were done without knowledge of survival or other clinical information. These determinations were also done without knowledge of the results of the other analyses.

### *Statistical analyses*

Survival analysis was done using a BMDP computer program (BMDP Statistical Software, Department of Biomathematics, University of California Press, Los Angeles, CA). Crude survival was estimated with the product-limit method, and comparison of survival between groups was done using the log-rank test (BMDP 1L). Frequency tables were analysed using the chi-square test or Fisher's exact test. The SPF distributions between weak and strong HR staining intensity groups were compared using Mann-Whitney's U-test. All $P$-values are 2-tailed.

### Results

Fourteen (52%) lymphomas were either negative or very weakly positive with Hermes antibody (HR −/+), nine (33%) showed moderate staining intensity (HR + +), and four (15%) were brightly positive (HR + + +, Figures 1–3). Fourteen of the 26 lymphomas with successful staining for LFA-1 beta were negative and 12 (46%) were positive.

Lymphomas with negative or very weakly positive CD44 expression were associated with more favourable survival than those with more positive CD44 expression (Figure 4). Only 15% of the patients with moderate or strong CD44 expression were alive 10 years after the diagnosis as compared with 57% if lymphoma cells did not express CD44 ($P = 0.02$). Of the other factors tested, patients with stage I lymphoma had better prognosis than those with stage II or IV lymphoma in a univariate analysis ($P = 0.04$), whereas DNA ploidy (diploid, $n = 16$, *vs* nondiploid, $n = 11$, $P = 0.25$), SPF ($\leqslant$median, 17%, *vs* $>$median, $P = 0.42$), presence of B-symptoms ($P = 0.34$), histological classification (MALT *vs* non-MALT, or low grade MALT lymphomas *vs* the rest, $P = 0.37$ and 0.30, respectively), sex ($P = 0.69$), or LFA-1 expression ($P = 0.71$) did not have significant association with survival. No significant association between CD44 expression (−/+ *vs* + + or + + +) could be found with sex, stage (I *vs* II or IV), DNA ploidy, SPF, LFA-1 beta expression, histological classification (MALT *vs* non-MALT, or low grade MALT lymphomas *vs* the rest), or the primary site (the stomach *vs* other, $P > 0.1$ for all comparisons).

### Discussion

Lack of CD44 expression was associated with favourable outcome in gastrointestinal lymphoma in the present series.

**Figure 1** Immunoblastic lymphoma with strong positive staining intensity (+ + +) for Hermes-3/CD44 in the lymphoma cells. A gastric gland in the upper left is unstained. The bar in the lower right corner is 75 μm.

**Figure 2** Centroblastic type of gastric lymphoma with intermediate (+ +) positive staining intensity for CD44 in the lymphoma cells. The bar is 75 μm.

**Figure 3** MALT lymphoma of low grade of malignancy with negative (+/−) staining for CD44 in the lymphoma cells. The bar is 75 μm.

Because CD44 expression was significantly associated neither with a large SPF, which is associated with aggressive histological features and poor outcome in lymphoma (Rehn *et al.*, 1990; Joensuu *et al.*, 1991), nor with high histological grade of malignancy, the result suggests that the poor outcome of CD44 positive gastrointestinal lymphomas may be due to their greater tendency to give rise to distant metastases. Although a multivariate analysis was not carried out due to the limited size of the series, in addition to CD44 expression only postsurgical stage showed some association with survival among the several factors studied, which suggests that lymphocyte CD44 expression may be one of the strongest prognostic factors in gastrointestinal lymphoma.

Fourteen (52%) of the gastrointestinal lymphomas did not express CD44. In a recent series consisting of 245 non-Hodgkin lymphomas investigated by us by similar methods (Jalkanen *et al.*, 1991) only 77 (31%) lymphomas were CD44 negative or expressed it weakly ($P = 0.03$), suggesting that gastrointestinal lymphomas may have a smaller tendency to

**Figure 4** Survival of 27 patients with gastrointestinal lymphoma by CD44 expression.

disseminate hematogenously than non-Hodgkin lymphoma in general. In accordance with this, gastrointestinal lymphoma may apparently occasionally be cured by local therapy, such as surgery alone (Dragosics *et al.*, 1985).

Several homing-associated molecules work in concert in lymphocyte extravasation. Therefore, a better correlation with survival might be obtained if a panel of these molecules were investigated, but analysis of most of such molecules is probably not possible from formalin fixed tissue. Lymphocyte adhesion molecule $\alpha 4\beta 7$ is likely to be involved in lymphocyte homing to Peyer's patches and the appendix (Hu *et al.*, 1992). Although little is known about lymphocyte homing receptors involved in the recruitment of immunoblasts or memory lymphocyte populations to the intestinal *lamina propria*, venules in the intestinal *lamina propria* express the mucosal vascular addressin, which appears to play an important role in recruiting gut-homing lymphocyte populations from the blood to this site. However, gut intraepithelial leukocytes and many *lamina propria* lymphocytes express the mucosal lymphocyte antigen (MLA), defined by MoAbs HML1 or Ber ACT8 in the human, which may play a role in lymphocyte homing to the gut (Picker & Butcher, 1992).

It is concluded that gastrointestinal lymphoma with absent or very weak CD44 expression is associated with more favourable prognosis than lymphoma with stronger CD44 expression. Larger series now need to be studied in order to investigate further the relationship between CD44 expression and different histopathological subtypes of gastrointestinal lymphoma, and between CD44 expression and other known prognostic factors in this disease. Studies performed from fresh lymphoma tissue where multiple homing-associated molecules are simultaneously evaluated are also highly warranted.

The study was supported by the Cancer Society of Finland, Turku University Foundation, and Sigrid Juselius Foundation.

### References

BARGATZE, R.F., WU, N., WEISSMAN, I.L. & BUTCHER, E.C. (1987). High endothelial venule binding as a prediction of the dissemination of passaged murine lymphomas. *J. Exp. Med.*, **166**, 1125–1131.

BUTCHER, E.C. (1991). Leukocyte-endothelial cell recognition: three (or more) steps to specificity and diversity. *Cell*, **67**, 1033–1136.

CAMPLEJOHN, R.S., MACCARTNEY, J.C. & MORRIS, R.W. (1989). Measurement of S-phase fractions in lymphoid tissue comparing fresh versus paraffin-embedded tissue and 4′,6′-diamino-2 phenylindole dihydrochloride versus propidium iodide staining. *Cytometry*, **10**, 410–416.

DRAGOSICS, B., BAUER, P. & RADASZKIEWICS, T. (1985). Primary gastrointestinal non-Hodgkin's lymphoma. *Cancer*, **55**, 1060–1073.

GALLATIN, W.M., BUTCHER, E.C. & WEISSMAN, I.L. (1983). A cell surface molecule involved in organ-specific homing of lymphocytes. *Nature*, **304**, 30–34.

HAMANN, A., JABLONSKI-WESTRICH, D., DUIJVESTIJN, A., BUTCHER, E.C., BAISCH, H., HARDER, R. & THIELE, H.G. (1988). Evidence for an accessory role of LFA-1 in lymphocyte-high endothelium interaction during homing. *J. Immunol.*, **140**, 693–699.

HEDLEY, D.W., FRIEDLANDER, M.L., TAYLOR, I.W., RUGG, C.A. & MUSGROVE, E.A. (1983). Method for analysis of cellular DNA content of paraffin-embedded pathological material using flow cytometry. *J. Histochem. Cytochem.*, **31**, 1333–1335.

HORST, E., MEIJER, C.J., RADASZKIEWICS, T., OSSEKOPPELE, G.J., VAN KRIEKEN, J.H. & PALS, S.T. (1990). Adhesion molecules in the prognosis of diffuse large cell lymphoma: expression of a lymphocyte homing receptor (CD44), LFA-1 (CD11a/18), and ICAM-1 (CD54). *Leukemia*, **4**, 595–599.

HU, C.T., CROWE, D.T., WEISSMANN, I.L. & HOLZMANN, B. (1992). Cloning and expression of integrin βp (β7): a functional role in Payer's patch -specific lymphocyte homing. *Proc. Natl Acad. Sci.*, **89**, 8254–8258.

ISAACSON, P. & WRIGHT, D.H. (1984). Extranodal malignant lymphoma arising from mucosa-associated lymphoid tissue. *Cancer*, **53**, 2515–2524.

JALKANEN, S., BARGATZE, R., LOS DE TOYOS, J. & BUTCHER, E.C. (1987). Lymphocyte recognition of high endothelium: antibodies to distinct epitopes of an 85-95 kD glycoprotein antigen differentially inhibit lymphocyte binding to lymph node, mucosal, or synovial endothelial cells. *J. Cell Biol.*, **105**, 983–990.

JALKANEN, S., JOENSUU, H., SÖDERSTRÖM, K.-O. & KLEMI, P.J. (1991). Lymphocyte homing receptor and clinical behavior of non-Hodgkins's lymphoma. *J. Clin. Invest.*, **87**, 1835–1840.

JOENSUU, H., KLEMI, P.J., SÖDERSTRÖM, K.-O. & JALKANEN, S. (1991). Comparison of S-phase fraction, Working Formulation, and Kiel classification in non-Hodgkin's lymphoma. *Cancer*, **68**, 1564–1571.

PALS, S.T., HORST, E., OSSEKOPPELE, G., FIDGOR, C.G., SCHEPER, R.J. & MEIJER, C.J.L.M. (1989). Expression of lymphocyte homing receptor as a mechanism of dissemination in non-Hodgkin's lymphoma. *Blood*, **73**, 885–888.

PALS, S.T., DEN OTTER, A., MIEDEMA, F., KABEL, P., KEIZER, C.D., SCHEPER, R.J. & MEIJER, C.J.L.M. (1988). Evidence that leukocyte function-associated antigen-1 is involved in recirculation and homing of human lymphocytes via high endothelial venules. *J. Immunol.*, **140**, 1851–1853.

PICKER, L.J. & BUTCHER, E.C. (1992). Physiological and molecular mechanisms of lymphocyte homing. *Annu. Rev. Immunol.*, **10**, 561–591.

PICKER, L.J., KISHIMOTO, T.K., SMITH, C.W., WARNOCK, R.A. & BUTCHER, E.C. (1991). ELAM-1 is an adhesion molecule for skin-homing T cells. *Nature*, **349**, 796–799.

PICKER, L.J., MEDEIROS, L.J., WEISS, L.M., WARNKE, R.A. & BUTCHER, E.C. (1988). Expression of lymphocyte homing receptor antigen in non-Hodgkin's lymphoma. *Am. J. Pathol.*, **130**, 496–504.

REHN, S., GLIMELIUS, B., STRANG, P., SUNDSTRÖM, C. & TRIBUKAIT, B. (1990). Prognostic significance of flow cytometry studies in B-cell non-Hodgkin lymphoma. *Hematol. Oncol.*, **8**, 1–12.

STANSFELD, A.G., DIEBOLD, J., NOEL, H., KAPANCI, Y., RILKE, F., KELENYI, G., SUNDSTRÖM, C., LENNERT, K., VAN UNNIK, J.A.M., MIODUSZEWSKA, O. & WRIGHT, D.H. (1988). Updated Kiel classification for lymphomas. *Lancet*, **i**, 292–293.